# Psychological care of patients during the pancreas transplantation process: issues and prospects

**DOI:** 10.3389/fcdhc.2023.1205964

**Published:** 2023-07-10

**Authors:** Vasiliki Galani, Orianne Villard, Valérie Olivier, Andrea Peloso, Philippe Compagnon, Fadi Haidar, Paco Prada

**Affiliations:** ^1^ Service of Liaison Psychiatry and Crisis Intervention, Department of Psychiatry, Geneva University Hospitals, Geneva, Switzerland; ^2^ Departement of Endocrinology, Diabetes, and Nutrition, Montpellier University Hospital, Montpellier, France; ^3^ Division of Nephrology and Hypertension, Department of Medicine, Geneva University Hospitals, Geneva, Switzerland; ^4^ Division of Transplantation, Department of Surgery, Geneva University Hospitals, Geneva, Switzerland

**Keywords:** pancreas transplantation, islet transplantation, time projection, psychological issues, treatment adherence, caregivers

## Abstract

Pancreas transplantation for patients with type 1 diabetes is a therapeutic option when other treatments are not effective and physical complications occur. Psychological burden is prominent in patients, and non-adherence to treatment is often one manifestation of such difficulties. Time projection is an important factor affected by chronic disease. The prospect of transplantation has the potential to repair this disruption. It could re-establish a continuity in the patient’s self and history, by connecting the future to a life that was only about past and present. Taking care of oneself, adhering to treatment, being part of a long-term therapeutic project and going through transplantation are all processes that need a good ability to self-project in time. This is specifically a domain of psychotherapeutic interventions. In this article, the psychological implications of pancreas transplantation for patients and caregivers alike will be discussed, as well as the role of the psychiatrist in the transplantation process.

## 1 Introduction

Affecting nearly 537 million people worldwide, diabetes is one of the most common chronic diseases. Whilst type 1 diabetes occurs at an early age with an autoimmune pathophysiology, type 2 diabetes is mostly associated with genetic and lifestyle risk factors and can be prevented or delayed in around 60% of cases (1, 2). However, long-term complications and their impact on patients’ life expectancy are crucial issues in the progression of both types of diabetes. As stated by Gonzalez et al, patients and their families are faced with the constant “challenge of integrating into their lives a demanding, complex and lifelong regimen to control their progressive illness and prevent or delay diabetes complications” (3).

Pancreas or islet transplantation are currently the only clinical solutions available and commonly accepted for an effective beta-cell replacement, being effective in achieving near-normal glycemic control. Selection of the best option for beta-cell replacement depends on several factors such as kidney function, patient comorbidities, and treatment goals. Simultaneous pancreas and kidney transplant (SPK) currently represents the treatment of choice for patients with type 1 diabetes and diabetic nephropathy. However, SPK is a major procedure in patients who often have multiple comorbidities. Other treatment options are available, including simultaneous islet kidney transplant, pancreas after kidney transplant, and islet after kidney transplant in patients with kidney disease, or pancreas transplant alone and islet transplant alone in patients without kidney disease (4). A functioning pancreas transplantation restores normal glucose homeostasis - decreasing severe hypoglycaemia and diabetes related complications occurrence - as well as improves quality of life and life expectancy (4, 5). After transplantation, insulin independence at 5 years is obtained in 50% to 80% of recipients (5, 6).

Regarding the psychological impact of the chronic aspect of diabetes, older studies offered a psychosomatic approach to the disease and were mostly concerned primarily with personality traits, family characteristics and life stress that might have an impact on the onset and course of diabetes (7). In the mid-1990s, the emotional impact of living with diabetes was further explored and the concept of “diabetes distress” was introduced into the literature to describe the “negative emotional or affective experiences resulting from the challenge of living with the demands of diabetes” (8, 9). Diabetes self-management and related psychosocial factors are still areas of research. However, we know that awareness of the disease and its treatment, the individual’s ability to regulate negative emotions, patients’ perceptions of self-efficacy and control over the disease, cultural beliefs, socioeconomic factors, and resilience factors may influence adherence to diabetes treatment and disease progression (3, 10, 11).

Personal future projection is also a factor that appears to be affected by chronic disease and can have an impact on diabetes treatment adherence. The prospect of pancreas transplantation potentially repairs this disruption and can restore continuity to the patient’s history and perception of the duration of time. The perception of time acts as a crucial unifying element of consciousness. It provides cohesion in the individual’s sense of self in a world of change. Lived time requires both the ability to remember and to wait (12). Enabling someone to regain the narrative articulation of his story and the perception of himself in time can be a psychotherapeutic challenge for both patients and their therapists during the pancreas transplantation process (13, 14).

## 2 Psychological aspects of diabetes diagnosis process

The diagnosis of diabetes can cause a disruption of time and set in motion the process of mourning for the person’s previous state of health. For some clinicians, this type of grief has particular characteristics because it is not only focused on losing external objects (activities, projects, relationships), but also on losing one’s body integrity and health. Patients have to face the painful experience of the irrevocable process of their illness and make the multi-daily adjustments to prevent acute and chronic complications.

Parallelly, diabetes represents the onset of a state of dependency on others and necessity of anticipation (treatment, monitoring, caregivers, the health care system, family). This transition can be a source of distress and a narcissistic failure for some patients. Dependency on others can lead to an accumulation of frustrations and alter the patient’s perception of time. In such cases, the patient’s sense of vulnerability may shift to grandiose compensation, expressed through a narcissistic retreat focused on the illness. In such cases, the therapeutic alliance with carers may be difficult to establish. Patients are most interested in their demanding illness on a daily basis and tend to neglect other areas of daily life, such as projecting into the future. This provides them with a new psychic equilibrium, which they tend to maintain as it allows them to experience a sense of regaining control over their body, self and personal history (15, 16). Addressing fear of dependency and maintaining the ability to rely on significant others remains a psychotherapeutic challenge in the treatment of people with diabetes.

It is important to remember that the patients’ and carers’ timelines may not coincide. The meaning of time changes and the fear of death increases. Perceptions of one’s own personal identity, relational and existential scope are reconsidered. Time is now experienced as a period of adaptation and acceptance of the new condition of the disease. Becoming aware of diabetes, understanding its underlying mechanisms and the treatment process, complying with medical follow-ups, but also finding a new balance in everyday life are some of the crucial adjustment points in diabetes self-management. The time spent caring for the disease can be perceived as “wasted” time during which patients are unable to care for themselves. Understanding these conditions in order to establish a solid therapeutic alliance is a major challenge for the carer (17).

The early onset of type 1 diabetes and its limitations in daily life, especially during the dynamic developmental process of adolescence, may be associated with difficulties in mastery on one’s body and self-care. Non-adherence is likely to be more common in young patients, who “have more difficulty imagining the person they want to protect through adherent behavior and for whom the disease will last longer” (18). For these patients, diabetes sometimes becomes a crucial part of their identity, influencing their life choices and their relationships with others. The transition to adulthood and independence may also be more complex for these patients (19). Parental stress may also create an insecure attachment model in diabetic children and adolescents and regulate their response to the demands of the disease (20).

The chronic process of diabetes freezes the patient’s perception of time in the present and gradually eliminates the notion of past and future. A repetitive pattern can emerge in the management of diabetes, following a specific regime, continuous monitoring of blood glucose levels and regular medical follow-ups. Although this pattern can lead to a more organized lifestyle, it can also lead to a loss of perspective. Patients may, based on their past experiences, disinvest and not engage in future projects to avoid interference with diabetes complications. In such cases, the lack of new experiences causes past and future to be confused, resulting in a disruption of lived time. Disruption of time projection can reduce motivation and lead to poor adherence to treatment (18, 21).

## 3 Pancreas transplantation and psychological issues

Pancreas or islet transplantation (intended as beta-cell replacement) is indicated in patients with type 1 diabetes who have problematic hypoglycaemia despite optimal medical therapy (22). Their principal aim is to provide a critical beta-cells mass able to restore endogenous insulin secretion and regulate blood glucose levels.

Pancreas transplantation can offer significant benefits to people with diabetes (5, 22), including:

1. improved blood glucose control, with the complete abolition of severe hypoglycemic episodes,
2. reduced reliance or even cessation on insulin therapy,
3. improved quality of life

With this in mind, the preliminary evaluation of transplantation must weigh the long-term benefits of such pancreas replacement against the morbidity and mortality associated with the surgery itself, the management of diabetes and the side effects of long-term immunosuppressive medications (23, 24). Scalea et al, demonstrated that patients who had undergone successful pancreas transplantation alone were mentioning better self-identified health scores and a better glucose control (25). The fear of hypoglycemia measured by the Hypoglycemia Fear Survey, as well as the 36-Item Short Form Heath Survey (SF-36) and the Diabetes Quality of Life (DQOL) were found to be improved after islet transplantation (26, 27). Compared to the whole pancreas transplantation, islet transplantation remains a less invasive technique and could be experienced by patients as a less stressful process. Nevertheless, the number of hospitalizations required may also be experienced as a chronic and demanding procedure. Comparing the pancreas and islet transplantation with regard to the health-related quality of life and psychological symptoms remains a field for further research (28).

The promise of pancreas transplantation for patients suffering from diabetes complications is an opportunity to improve their daily lives. The potential to be less dependent on insulin treatment and to prevent diabetes complications opens up a new and promising time horizon that has been limited in the past. Reducing the burden of diabetes restores the natural flow of time and repairs the disruption of (vital) becoming; a concept first described in mental disorders (14, 29, 30). The construction of new projects becomes possible again and participation in new plans is reconsidered. Patients are often positively involved during the preliminary assessment and medical examinations to become eligible for transplantation. They also note a better sense of time and a reduction in depressive symptoms and anxiety.

Whilst the pre-transplantation evaluation phase is often experienced as a particularly exciting, dynamic and constructive process, the concretization of the therapeutic plan in the case of eligibility is often followed by a period of distress. The indefinite period of waiting for the operation, feelings of impatience, discouragement and anxiety, including worries about the donor’s death, the graft’s engraftment and the necessary post-transplant medical regimen, create uncertainty and raise questions about the healing effect of the transplant (31, 32). Communicating these concerns and uncertainties to members of the transplantation team is often hampered by patients’ fears that such discussions will affect their eligibility for transplantation (33).

Patients may face practical difficulties in materializing their future projects, as the chronic progression of diabetes and its complex demands in everyday life affect their ability to deal with long-term goals which are unrelated to diabetes and their health problems. In addition, the prospect of transplantation may be experienced as a threat to their psychological equilibrium built up over years of illness. This state of distress is sometimes expressed in patients’ acting out behavior. Missed medical appointments and poor management of the disease, leading to hospitalization, may jeopardize their suitability for transplantation. In such cases, consultation-liaison psychiatrists are asked to reassess the patients’ motivation, potential for compliance after surgery, and the presence of emerging adjustment, mood or anxiety disorders. We believe that underneath this implicit caregiver demand, there is an explicit demand for psychotherapeutic treatment to support patients undergoing transplantation (34).

Suicidal ideation, self-harm and suicide risk are also found to be increased among patients suffering from diabetes mellitus and comorbid mood disorders. Diabetic patients have a three to four times higher risk to attempt suicide than the general population. Feelings of hopelessness related to chronic disease, impaired quality of life and accessibility to potential lethal drugs as insulin are only some factors that have an impact on the complex relationship between suicide and diabetes (35, 36). Suicide risk is also more prevalent among transplant candidates and recipients than in the general population and patients who undergo pancreas transplantation tend to be at higher risk (37, 38). Marfil-Garza et al. stated that 16% of post islet transplantation recipients’ deaths in their cohort were due to suicide (39). These findings highlight the importance of the preliminary psychological assessment as well as the need for a continued psychological support for patients who undergo pancreas or islet transplantation.

The psychotherapeutic setting, through its consistency and regularity, and the presence of a stable interlocutor, the therapist, can create a safe space. During the sessions, we explore the patient’s current experiences, the interference of diabetes symptoms in daily activities, the difficulties in coping with the unpredictable aspects of the disease, but also the patient’s personal resources and skills developed through this chronic process. Starting from the present and using a basic stance of curiosity and empathic validation, an understanding of the patient’s emotional state may become possible and the patient’s reflexivity may be enhanced (40, 41).

During the sessions, past experiences are reviewed and goals for the foreseeable future are discussed with the patient in order to serve as realistic time projections. Achieving short-term goals and receiving positive feedback can strengthen their self-esteem and help to gradually regain a sense of control. Facing and coping with triggering situations allows patients to gain a better understanding of themselves and their resources (42). Gradually, the patient’s projection into the long-term future may become more realistic. We hypothesize that pancreas transplantation can then be seen as a means to achieve well-being. Being less idealized, this project could lose its potentially threatening character and be carried out under better conditions.

Regular psychotherapeutic sessions, combined with routine medical follow-ups, might help to build a more solid alliance with caregivers, who will be considered the patient’s “partners”. Connection with others can be experienced as an opportunity to improve quality of life and reduce fear of dependency. In this way, we believe that patients can potentially regain autonomy and control over their history. Past, present and future tend to form a whole, and skills of remembering and waiting may be developed, helping patients to cope with the temporalities of diabetes.

The integration of psychotherapeutic sessions into medical follow-ups after transplantation is also recommended, as this transitional period may be characterized by an increased prevalence of psychological symptoms that affect patients’ quality of life (43, 44). Gibbons et al. support that improved treatment satisfaction, well-being, self-reported health, general quality of life and less negative impact on renal-specific quality of life are observed after simultaneous pancreas and kidney transplantation, but unrealistic pre-transplantion expectations may cause some disappointment (45). We believe that continuing psychological support during this adjustment period could help patients through the changes that follow pancreas transplantation in terms of physical and metabolic needs, body image, relating to a new medical team and coping with immunosuppressive treatment. Psychological support is also useful to help patients cope with changes in self-perception, perspective, family, work and relationship dynamics (46). Attention to the psychological aspects of pancreas transplantation remains a key issue for clinicians to contribute to optimal pre- and post-transplant care.

## 4 Conclusions

Diabetes constitutes a demanding and complex chronic disease that affects patients’ quality of life. Despite the remarkable progress in diagnosis and treatment means, there is evidence of various barriers to an efficient control and optimal health outcomes for patients with diabetes (47). Factors with impact on diabetes management can be related to both patients’ and caregivers’ attitudes. Mental health disorders do often interfere with diabetes management behaviors and tend to increase morbidity and poor adherence to treatment (48). Addressing diabetes distress remains a challenge in clinical practice (49, 50).

Psychological burdens and difficulties in time projection are often observed when taking care of diabetic patients. Repetitive patterns during diabetes management and avoidance of future projects are often described and can contribute to patients’ altered perception of lived time.

Pancreas or islet transplantation is a challenging option that can open up patients’ time horizons and improve their daily lives. Waiting for a transplant is often experienced as an exciting transitional period, but feelings of fear, impatience, anxiety, and discouragement may also arise. Psychotherapeutic interventions before and after transplantation can help patients cope with the changes associated with the prospect of transplantation and prevent possible adverse effects before and after surgery. Being aware of these psychological aspects remains a key point for clinicians involved in the transplantation process.

## Data availability statement

The original contributions presented in the study are included in the article/supplementary material. Further inquiries can be directed to the corresponding author.

## Author contributions

VG and PP contributed to the conception of this article. All authors contributed to manuscript revision, read, and approved the submitted version.
